# Asylums of the World


**Published:** 1892-07-09

**Authors:** 


### INSTITUTION LITERATURE.

### ASYLUMS OF THE WORLD.*

#### II.—TREATMENT.

The author devotes one portion of "Asylums of the World" to contrasting the present effect of public opinion upon the great question of the treatment of the insane with former periods, and shows how modern lunatic asylums have improved. The almost total abolition of mechanical restraint, except in surgical cases and in cases of exceptional violence, is in itself sufficient proof that in Great Britain, at any rate, the system of force and repression has been superseded by more effective and more advanced methods. The substitutes which the modern system have advanced for the replacement of physical force in the treatment of the insane are their rational and constant employment, a greater amount of personal liberty, securing, where that is possible, the individuality of the patient, and supplying, from time to time, a carefully-selected series of amusements and recreations. These advantages, in combination with the medical treatment of acute cases, form the basis of the great system of asylum management which, to their credit, the alienist

* "Hospitals and Asylums of the World." By Henry C. Burdett Vols. I. and II.—Asylums and Asylum Construction. (London: J. and A. Churchill.)

physicians of the latter part of the nineteenth century have succeeded in establishing. Notwithstanding the great advances made in recent years, we are not prepared to agree entirely with the writer that the present system generally adopted in this country, of herding in one large asylum all classes and conditions of lunatics is by any means the best that can be discovered. There are one or two evils arising out of the system which the writer himself recognises. The first of these is the evil of overcrowding. The evils are apparent and must be admitted by all, and yet the cry goes up from almost every asylum in our land "we are overcrowded." The voice of public opinion seems to have been lulled chiefly, we think, because it feels that a great duty has been done by relieving the general public of a social inconvenience by confining all lunatics in the asylums provided for that purpose. Further scientific enlightenment demands free scope for the medical treatment of the acutely insane. With the view of retaining as much as possible of what is really good in the existing régime an admirable proposal is made which we have since heard publicly advocated. It is that the population of each asylum should be limited to 1,000, that the asylum should contain a central separate hospital for the treatment of acute cases of mental and bodily disease, wherein the medical and nursing instincts of the staff would be trained and elevated; around this central hospital would be disposed the other parts of the asylum so constructed and arranged as to be suitable for the reception of the various classes of chronic patients. It is with satisfaction that we record here that the new Scottish asylums and some of the older ones are being modelled on this principle. From this step, which we think in the right direction, every conceivable scheme, each of them having for its sole object the purpose of the better medical treatment of the acutely insane, and the closer union between lunacy practice and other forms of medical science has taken its origin. Ultimately, indeed, we find men sincerely advocating that every large hospital attached to a medical school should have its wards for the more scientific treatment of insanity, and for the instruction of medical students in a very important branch of their profession.

"In Asylums of the World" a pretty picture is given of the new asylum at Kankakee. For a description of the proposed lunacy hospital which it was intended to establish in London, the reader is referred to a voluminous appendix in the second volume of this work, where the evidence of experts before the Committee of the London County Council is given *in extenso*.

A review of asylums in Great Britain and Ireland is also given. The lunacy systems in England and Scotland, though in many points dissimilar, offer, on the whole, very few distinctive differences that are not founded upon the different codes of law of the respective countries, as well as upon the habits of the people. Looking, first of all, to the accommodation provided for the richer class of patients, we find both in England and Scotland that ample provision is made in private asylums. Registered hospitals and licensed houses in England and in the private and Royal asylums in Scotland are provided for the accommodation of patients paying the higher rates of board. A few patients, whose friends are able to afford only the lower rates of board, are accommodated in the county asylums in England and the district asylums in Scotland. It is a regretable fact that up to the present time no adequate provision has been made in either country for the reception in asylums of a class of patients to whom the associations of a pauper asylum are revolting, and who are yet unable to afford to pay the rate of board which would entitle them to the privileges of a better class asylum. It appears to us that the French system, which provides for the reception of paying patients into special wards of the provincial asylums, is an admirable one, and one that might, with mutual advantage to paying patients and asylums, be adopted in England and Scotland. The boarding-out of the insane in Scotland is a system so far in excess of what in England is called out-door pauper lunatics that it deserves a few passing comments. We are told that there are 2,573 insane persons boarded out in the houses of the peasantry throughout Scotland. Most of these patients have been in asylums, but having been found to be incurable and incapable of deriving benefit from a continued stay there, they have been transferred to the houses of those willing to take their care. We learn that the average daily cost of he maintenance of pauper lunatics in private dwellings, including clothing, is 10½d. per day. We believe that the boarding-out system forms an excellent outlet for the chronic harmless insane population which would otherwise accumulate in the existing asylums. The patients, as a rule, have the advantage of home life. They are generally well cared for, and as a proof of the salubrity of the system, it is stated that the death-rate of those so domiciled is only 4·5 per cent., or a little more than half the death-rate of asylums. It is right, however, to qualify such a statement by the admission that the boarded-out patients are a specially-selected class. We have no desire to criticise adversely a system which has hitherto been fraught with incalculable benefit to a large section of the insane in Scotland. It is manifestly unsuited for adoption throughout the greater part of England, and if it is to continue to survive in Scotland, it is our opinion that it must be modified so as more and more to resolve itself into colonies throughout the kingdom for the feeble-minded and the industrious insane. We do not for a moment suggest the idea that such colonies should ever model themselves after the example of Gheel, near Antwerp, but that the tendency of the boarding-out system, if it survives, will be in such a direction we not only believe but for the good of the insane we also hope. At page 422 we are given a charming picture of some of the insane colonies in Germany at Enium, for instance, which is an agricultural colony of 350 acres; there are 80 patients who work the farm. The dwelling-house is a model of what a combined farmhouse and modern asylum ought to be, and the whole place is under the management of a head attendant or steward, who is directly responsible to the authorities of the Hildesheim Asylum.

The labour of the insane now forms one of the most important curative agencies with which the modern alienist is acquainted. At the same time there are many patients who grumble at having to work without pay. We are particularly glad to see that the money payment of patient's labour is a question that is seriously discussed by the writer. In France such a system has been in vogue since the year 1857. When a patient is placed in a French asylum it is the duty of a provisional administrator, without waiting for an application to act, to institute an enquiry into the patient's means and goods, and to preserve them for him intact until his discharge. On his discharge from the asylum each patient receives a grant, or *peculium*, equivalent to 8s. or 10s., with the object of enabling him to resume his employment. As this sum is considered insufficient, patronage societies have been established throughout the country, with the object of supplementing this small *peculium*. For, as we are told at page 351, people mistrust individuals who have been discharged from lunatic asylums, and are afraid to trust them with tools, and only too frequently the privations and mortifications which they suffer bring about a relapse. We are glad to observe that a society for the aid of discharged lunatics is being formed in the metropolis. At the same time it seems hard that a patient who has laboured for months, it may be years, for the improvement of the asylum in which he has been lodged should be discharged without any remuneration for his past labours often without a respectable suit of clothing, and, most frequently of all, without a helping hand, to aid him in a cold and hostile world.

